# Treatment of immune checkpoint inhibitor-induced bullous pemphigoid with methotrexate

**DOI:** 10.1016/j.jdcr.2024.09.019

**Published:** 2024-10-11

**Authors:** Emma L. Myers, Paul B. Googe, Donna A. Culton

**Affiliations:** a University of North Carolina School of Medicine, Chapel Hill, North Carolina; b Dermatopathology Laboratory, Department of Dermatology, School of Medicine, University of North Carolina at Chapel Hill, Chapel Hill, North Carolina; c Department of Dermatology, School of Medicine, University of North Carolina at Chapel Hill, Chapel Hill, North Carolina

**Keywords:** bullous pemphigoid, cutaneous immune-related adverse effects, immune checkpoint inhibitor-induced bullous pemphigoid, immunotherapy, methotrexate, oncodermatology

## Introduction

Bullous pemphigoid (BP) is an autoimmune blistering disorder typically characterized by pruritic tense bullae caused by autoantibodies targeting the hemidesmosomal proteins BP180 and BP230.^1^^,^^2^ Immune checkpoint inhibitors (ICIs) are being increasingly utilized in the treatment of many malignancies; however, cutaneous immune-related adverse effects (cirAEs) are common.^1^ ICI-induced BP (ICI-BP) is a cirAE that affects 1.0% of ICI-treated patients.^1^^,^^3^ When left untreated, ICI-BP may lead to significant morbidity. In addition, immunosuppressive systemic therapies used to treat ICI-BP and/or ICI interruption required to adequately control ICI-BP may result in a worsening of the cancer prognosis.^3^ As a result, ICI-BP represents a particularly challenging cirAE in both oncological and dermatologic care and the identification of therapeutic agents that manage the skin toxicity with minimal immunosuppression is of particular interest.

As a steroid-sparing, cost-effective, and widely available immunomodulator, methotrexate has demonstrated efficacy for ICI-induced psoriasis, BP unrelated to ICIs, and rheumatologic irAEs.^4^ Yet, methotrexate use in ICI-BP remains infrequently described.^4^ This retrospective review of 4 methotrexate-treated ICI-BP patients showed that all patients achieved BP resolution, discontinuing steroids on average 4.0 months after starting methotrexate. Methotrexate was well-tolerated; only 1 patient (25%) experienced mild side effects (fatigue, gastrointestinal upset). These cases and a review of similarly published cases highlight methotrexate as a safe, effective treatment for ICI-BP, minimizing prolonged high-dose steroid exposure.

## Methods

We conducted a retrospective case review of 4 patients with ICI-BP, all of whom were diagnosed by routine histopathology and immunofluorescence and received care from the University of North Carolina Dermatology department over the last 3 years. Patient demographics, detailed oncological histories, and the characteristics of ICI-BP were compiled through review of medical records. We assessed the tolerability and therapeutic efficacy of methotrexate in managing ICI-BP cases triggered by the administration of nivolumab (*n* = 2) and pembrolizumab (*n* = 2). To augment our case series findings, we performed a literature review to identify additional instances where methotrexate was employed in the treatment of ICI-BP. This inquiry allowed us to compare our observations with reported outcomes, thereby providing a broader context and deeper insights into the management of ICI-BP with methotrexate.

## Results

The interval between the initiation of ICI therapy and biopsy-proven BP onset averaged 16.0 months, with a range of 11.9 to 22.0 months. Patients were treated with methotrexate for a duration ranging from 8.9 to 40.4 months, with dosages adjusted to 7.5-15 mg weekly based on tolerance and clinical response. All patients (*n* = 4, 100%) remained on ongoing therapy with methotrexate at the time of manuscript submission, as indicated in the most recently documented clinic note.

Prior to receiving methotrexate, all patients trialed various ICI-BP treatments without resolution, including prednisone, intravenous immunoglobulin, dapsone, niacinamide, doxycycline, and topical steroids. All prior systemic treatments were discontinued upon initiation of methotrexate with the exception of prednisone. Following the transition to methotrexate therapy, all patients (*n* = 4, 100%) achieved resolution of BP signs and symptoms and were able to discontinue systemic steroids after an average duration of 4.0 months (ranging from 2.3 to 5.8 months). Post-treatment, only one patient exhibited a mild, localized BP flare, which was effectively managed with topical corticosteroids alone. The onset of ICI-BP resulted in immunotherapy cessation in all cases. Cancer status following ICI discontinuation and BP treatment varied, including one patient with ongoing complete remission, 2 maintaining stable disease, and one experiencing disease progression (Table I).

**Table I:** ICI-BP treated with MTX at the University of North Carolina (UNC) and summary of cases in literature

	UNC dermatology				Shi et al^4^					Rofe et al^5^
Patient	1	2	3	4	5	6	7	8	9	10
Age	79	76	79	76	N/A	N/A	N/A	N/A	N/A	N/A
Sex	M	M	M	F	N/A	N/A	N/A	N/A	N/A	N/A
Primary cancer	Melanoma	SCC (lung)	RCC	SCC (tongue)	Melanoma	Melanoma	Lung	Melanoma	RCC	Melanoma
ICI	Nivo	Pembro	Nivo	Pembro	Pembro	Pembro	Nivo	Pembro	Nivo	Pembro
Time to BP-onset (mo.)∗	11.9	22	12.8	17.3	10	19	14	7	19	7
Diagnosis	Bx, DIF	Bx, DIF	Bx, DIF	Bx, DIF	Bx, Titers	Bx	Titers, Clinical†	Bx	Titers, Clinical	Bx, DIF, ELISA, Titers
Tx prior to MTX‡	Pred, dox; dap; TCS	IVIG; dox; nam; TCS	Pred; dox; TCS	Pred; TCS	Pred; MMF; dap; dox; nam; TCS	Pred; mino; TCS	Pred; TCS	None	TCS	Pred; TCS
MTX dosage (mg weekly)	15	15	7.5	10	25	10	15	10	10	20
MTX duration (mo.)§	40.4	13.3	8.9	13.9	(A) 6‖ (B) 9	8	48	10	5	N/A (ongoing)
Concurrent pred	Yes	Yes	Yes	Yes	(A) Yes‖ (B) No	Yes	Yes	No¶	No	Yes
Able to discontinue pred on MTX?	Yes	Yes	Yes	Yes	Yes	Yes	Yes	-	-	N/A
MTX start to pred d/c (mo.)	2.3	5.8	2.9	4.9	(A) Continued with MTX (B) N/A	2	18	-	-	N/A
Dz exac. upon pred d/c	No	No	No	Local flare	No	No	No	-	-	N/A
AE to MTX	No	No	No	Yes#	No	Yes#	No	No	No	No
ICI-BP response to MTX∗∗	Res	Res	Res	Res	Res	Res	Res	Res	No improv	Improv
ICI cessation d/t ICI-BP	Yes	Yes	Yes	Yes	Yes	Yes	Yes	Yes	Yes	Yes
Cancer status after ICI-BP	Stable	CR	Stable	Prog.	Prog.	Stable	Stable	CR	Stable	CR

*AE*, Adverse effect; *Bx*, biopsy; *CR*, complete remission; *CTCAE*, Common Terminology Criteria for Adverse Events; *DIF*, direct immunofluorescence; *Dap*, dapsone; *Dox*, doxycycline; *Dz*, disease; *ELISA*, enzyme-linked immunosorbent assay; *Improv*, improvement; *IVIG*, intravenous immunoglobulin; *Min*, minocycline; *Mo*., months; *MTX*, methotrexate; *N/A*, not applicable; *Nam*, niacinamide; *Nivo*, nivolumab; *Pembro*, pembrolizumab; *Pred*, prednisone; *Prog*., progression; *RCC*, renal cell carcinoma; *Res*, resolution; *SCC*, squamous cell carcinoma; *TCS*, topical corticosteroids; *Tx*, therapy.

∗ Time between initiation of ICI therapy and BP onset.

† Patient declined skin biopsy.

‡ All prior systemic treatments were discontinued upon initiation of MTX with the exception of prednisone.

§ Defined as time from MTX initiation to manuscript submission (5/6/2024); all patients (*n* = 4) on ongoing therapy with MTX at time of manuscript submission, as indicated in the most recently documented clinic note.

‖ MTX was initially given concurrently with prednisone for 2 months, then stopped after 6 months due to complete improvement. Recurrence occurred 6 months later; MTX alone achieved complete improvement.

¶ Patient had previous ICI-induced adrenal insufficiency, maintained on 5 mg prednisone daily, with no additional steroids needed for ICI-BP control.

# AE did not require discontinuation of MTX.

∗∗ Shi et al^4^ assessed ICI-BP severity based on CTCAE. Thus, resolution defined as no evidence of disease or post-MTX CTCAE score of 0 and lack of improved defined as no change in pre- to post-MTX CTCAE score.

## Discussion

ICI-BP occurs in 1% of patients receiving anti-programmed cell death protein 1 (PD-1) and anti-PD-1 ligand (PD-L1) agents. This prevalence is notably higher when compared to idiopathic BP, which affects only 0.005% of the general population, highlighting the significant toxicity induced by ICIs.^1^, ^2^, ^3^ The underlying mechanisms of ICI-BP are not fully understood; however, it is presumed to be associated with the activation of PD-1 and PD-L1 expressing self-reactive T cells and B cells.^4^ Early recognition and management of ICI-BP is necessary to reduce the morbidity associated and impact on cancer treatment; however, delays in diagnosis and adequate treatment are common due to differences in clinical presentation, histologic findings, and treatment responses between idiopathic and ICI-BP.^1^

**Fig 1:**
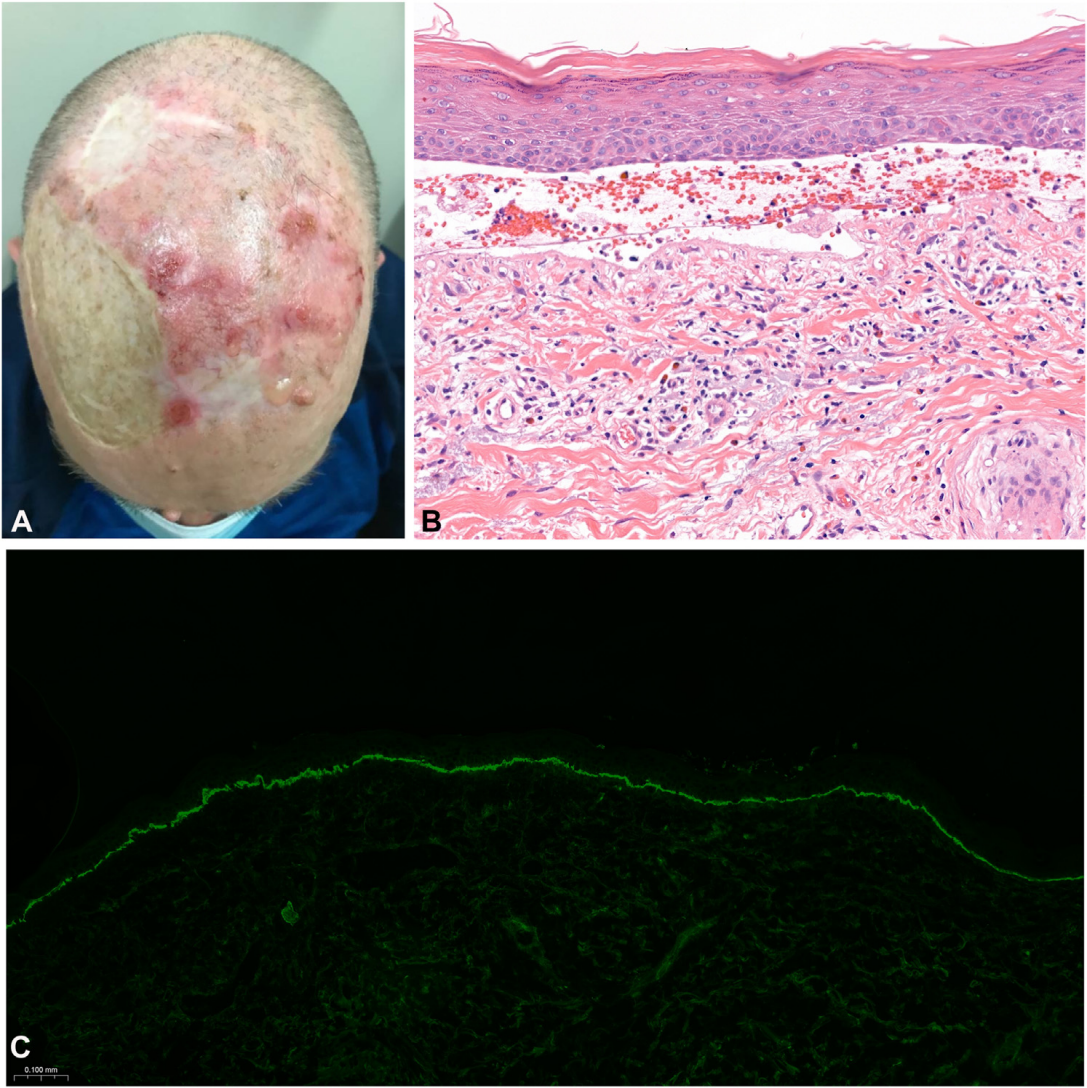
Clinicopathological findings of a representative patient (patient 1). **A,** Tense bullae with adjacent hemorrhagic erosions on scalp. **B,** H&E staining at 40× magnification of scalp biopsy lesion showing subepidermal vesicle with lympho-eosinophilic infiltrate. **C,** DIF at 10× magnification from perilesional punch biopsy with positive linear deposits of C3 (and IgG not shown) at the basement membrane zone. *C3*, Complement component 3; *DIF*, direct immunofluorescence; *H&E*, hematoxylin and eosin; *IgG*, immunoglobulin G.

**Fig 2:**
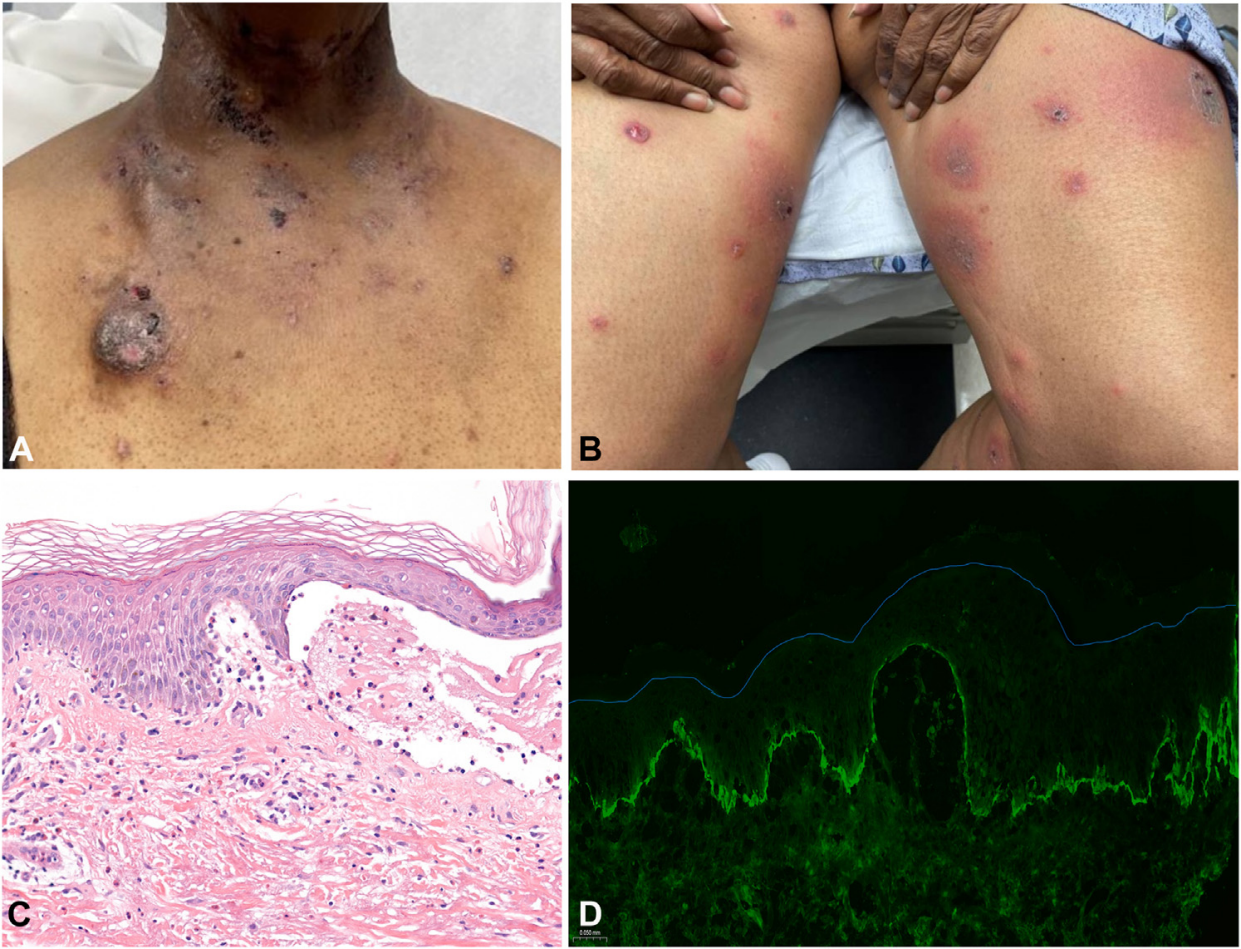
Clinicopathological findings of a representative patient (patient 4). **A** and **B,** Scattered erythematous vesicles, tense bullae, and crusted erosions over chest including port site (**A**) and bilateral lower extremities (**B**). **C,** H&E staining at 40× magnification from left arm punch biopsy showing subepidermal bullous dermatosis with papillary dermal edema and superficial dermal perivascular lympho-eosinophilic infiltrate. **D,** Direct immunofluorescence (DIF) at 20× magnification from perilesional punch biopsy of left arm with positive linear deposits of C3 (and IgG not shown) at the basement membrane zone with blue line demarcating epidermis. *C3*, Complement component 3; *H&E*, hematoxylin and eosin; *IgG*, immunoglobulin G.

Compared to idiopathic BP, ICI-BP may affect a younger patient demographic and is more likely to present without the classic tense bullae seen in idiopathic BP (Figs 1, *A* and 2, *A* and *B*).^6^^,^^7^ Furthermore, ICI-BP cases typically demonstrate a significantly longer preceding interval of rash-free pruritus as well as more time between initial symptom onset (pruritus or cutaneous lesions) and diagnosis.^7^ This delay occurs despite similar intervals from symptom onset to dermatology referral in both idiopathic and ICI-BP.^7^

The diagnostic approach in ICI-BP is similar to idiopathic BP with biopsies for both hematoxylin and eosin and direct immunofluorescence (DIF) necessary to establish a firm diagnosis. However, compared to idiopathic BP, ICI-BP is less likely to show a subepidermal separation on histopathology and may have nonspecific findings such as intraepidermal vesicles, necrotic keratinocytes, a prominent eosinophilic infiltrate, and occasional thrombus formation (Figs 1, *B* and 2, *C*).^6^^,^^7^ The diagnosis of both idiopathic and ICI-BP is confirmed by DIF demonstrating linear deposits of immunoglobulin G and complement component 3 along the basement membrane zone (Figs 1, *C* and 2, *D*).^1^^,^^2^ Serological tests, such as indirect immunofluorescence and enzyme-linked immunosorbent assay, which may detect anti-BP230 or anti-BP180 antibodies, are useful in diagnosing BP.^8^ There are no significant differences in DIF or serological positivity in ICI-BP compared to idiopathic BP.^6^^,^^7^^,^^9^ Interestingly, the levels of anti-BP180 immunoglobulin G have been linked with improved therapy response and overall survival, as well as a higher propensity of developing ICI-BP during anti-PD-1/PD-L1 treatment in patients with non-small cell lung cancer.^9^ Lastly, marked eosinophilia in serum is more frequently observed at the time of initial rash presentation in ICI-BP cases, as compared to idiopathic BP.^6^^,^^7^

ICI-BP typically requires ICI treatment to be held and often permanently discontinued.^6^ Current treatment algorithms for ICI-BP are similar to idiopathic BP and recommend a therapeutic ladder moving from topical steroids to systemic corticosteroids to immunosuppressive treatments such as azathioprine, mycophenolate, and rituximab; however, the clinical impact of immunosuppression on the ICI-mediated antitumor response remains unknown and optimal treatment is not clearly defined.^7^ Studies have shown that ICI-BP is more likely to require systemic corticosteroids, more likely to require systemic immunosuppressives, and more likely to fail multiple systemic medications (including non-immunosuppressive options such as doxycycline and niacinamide) compared to idiopathic BP.^6^^,^^7^ Dupilumab, which blocks interleukin-4Rα, has shown promise for treatment of ICI-BP in several case studies given its excellent safety profile and lack of immunosuppression.^10^^,^^11^ There are, however, some reservations in treating ICI-BP with dupilumab due to its specific suppression of the type 2 inflammatory pathway in light of current data indicating the advantageous impact of type 2 responses, especially eosinophilia, on the efficacy of ICI therapies.^12^ Therefore, treatment with a reliable safety profile, minimal immunosuppression, and a promising mechanism of action is needed.

Indirect inhibition of antitumor-specific T cells is favorable to avoid hindering the ICI antitumor effect. Unlike other immunosuppressive agents such as mycophenolate mofetil and azathioprine, methotrexate does not directly inhibit T-cells targeting underlying cancer, theoretically reducing the risk of tumor progression.^13^ Methotrexate has demonstrated benefits for patients with idiopathic BP as both a well-tolerated and effective therapeutic option. Studies have demonstrated that methotrexate, when used as a low-dose monotherapy or in combination with systemic corticosteroids, achieves clinical remission rates ranging from 43% to 100% and 35% to 60%, respectively, in idiopathic BP.^14^^,^^15^ These data suggest that methotrexate can be an advantageous strategy for clinicians aiming to minimize the use of systemic steroids and thereby reduce steroid-related side effects and associated morbidity and mortality risks.^15^ However, the use of methotrexate specifically in the context of ICI-BP remains underexplored, with limited cases reported in the literature.^4^^,^^5^

Our cohort highlights 4 cases of ICI-BP successfully treated with methotrexate resulting in remission, with only one patient experiencing a localized flare that resolved with topical steroids. Methotrexate was well-tolerated, with a single occurrence (*n* = 1, 25%) of fatigue and gastrointestinal upset (*n* = 1) that did not necessitate cessation of therapy. All patients had previously tried various treatments, which either led to minimal improvement or disease exacerbation upon steroid tapering. Methotrexate allowed for successful taper off steroids in all 4 patients underscoring its efficacy as a steroid-sparing alternative.

Literature review revealed 6 cases of ICI-BP treated with methotrexate. In a case series of 5 patients by Shi et al, most experienced complete resolution (*n* = 4, 80%) and one patient experienced no improvement of symptoms (*n* = 1, 20%).^4^ All patients who were concurrently managed with systemic steroids (*n* = 3, 60%) were able to discontinue systemic steroids while on methotrexate without disease exacerbation.^4^ One of these 3 patients was already on chronic low-dose prednisone for noncutaneous irAE but did not require dose adjustment for ICI-BP.^4^ The remaining 2 patients did not receive concurrent systemic steroids, one of which was the only case noted in the literature review without any improvement of their ICI-BP in response to methotrexate.^4^ Only one (20%) had adverse effects (mild diarrhea), not necessitating treatment discontinuation. A case report by Rofe et al highlighted one patient with significant improvement and only occasional single blisters after treatment with methotrexate.^5^ This patient was also concurrently managed with systemic steroids, although the time to steroid taper or discontinuation is unknown.^5^ Cancer status after ICI-BP varied among cases reported in the literature, with outcomes including tumor progression (*n* = 1), stable disease (*n* = 3), and complete remission (*n* = 2), indicating that further investigation is required to better capture the effects of methotrexate for ICI-BP on oncologic outcomes.

All 6 previously reported ICI-BP cases treated with methotrexate showed a similar pattern of efficacy, with most patients achieving disease resolution (*n* = 4 of 6) without significant adverse effects. Notably, most patients were on concurrent steroids (*n* = 4 of 6), and most (*n* = 3 of 4) were able to discontinue systemic steroids while on methotrexate without worsening ICI-BP (the other case was still on concurrent prednisone with ongoing methotrexate therapy at the time of publication^5^). No patients (*n* = 3 of 3) experienced disease exacerbation upon prednisone discontinuation, suggesting reduced systemic steroid dependence with methotrexate.

Given that ICI-BP led to ICI discontinuation in all cases (both at our institution and in the literature, *n* = 10), the higher degree of immunosuppression required for treatment compared to standard BP ^7^ and the negative effects of ICI-BP on health, it is clear that effective ICI-BP treatments are needed. Our findings indicate that methotrexate is effective and safe in inducing clinical improvement in ICI-BP and minimizing prolonged high-dose systemic corticosteroid exposure, and therefore should be considered in ICI-BP treatment guidelines.

The limitations of this study include the small sample size and its retrospective nature of the cohort study. Prospective studies with larger patient populations are needed to better delineate the role of methotrexate in treating ICI-BP and evaluate its influence on the long-term effects on tumor progression.

## Conflicts of interest

None disclosed.
